# Leiomyosarcoma after Total Laparoscopic Hysterectomy with Power Morcellation

**DOI:** 10.1155/2019/9381230

**Published:** 2019-10-02

**Authors:** Tsukasa Takahashi, Tomohisa Ugajin, Noriaki Imai, Atsushi Hayasaka, Nobuo Yaegashi, Takeo Otsuki

**Affiliations:** ^1^Department of Obstetrics and Gynecology, Tohoku University Hospital, Sendai, Miyagi, Japan; ^2^Department of Obstetrics and Gynecology, Sendai City Hospital, Sendai, Miyagi, Japan

## Abstract

**Introduction:**

Power morcellation is an effective and minimally invasive technique used to remove specimen tissues or the uterus in total laparoscopic hysterectomy (TLH). However, it has the risk of intraperitoneal dissemination of tissue and can cause a parasitic myoma. We report a case of leiomyosarcoma that occurred 4 years after TLH with power morcellation for fibroids.

**Case:**

A 52-year-old woman was referred to our hospital with a pelvic mass. She was diagnosed to have submucosal fibroids and had undergone TLH with power morcellation 4 years previously. The uterus weighed 398 g at that time. At present, a parasitic myoma was suspected, owing to the diagnosis of fibroids on the initial pathological evaluation. She underwent laparotomy, and the tumor was removed. Although the pathological evaluation confirmed the tumor to be a leiomyosarcoma, a review of the initial tissue did not show the presence of any malignancy. Since there was no metastasis, she was followed-up without additional treatment.

**Conclusion:**

Even if the initial pathologic evaluation suggests a benign mass, parasitic myoma and even sarcoma can occur after TLH with power morcellation. Considering the risk of dissemination and occult malignancy, the use of power morcellation should be avoided if there are alternative options to remove the tumor.

## 1. Introduction

The techniques for minimally invasive surgery (MIS) have dramatically improved in recent years, and the American College of Obstetrician and Gynecologists recommends MIS for the treatment of benign gynecologic diseases [1]. Sometimes, the tissue specimen for extraction from the abdominal cavity or the uterus may be too large to pass intact through the access ports or the vagina, and the surgeon morcellates the specimen into smaller fragments manually using a surgical scalpel or with a power device called a power morcellator. Although power morcellators allow for faster removal of uterine tissue, they can disseminate the fragments of specimen such as leiomyoma and endometriosis, which may implant on abdominal organs, causing inflammation, infection, and intestinal obstruction. Parasitic myomas are defined as disseminated leiomyomas that receive their blood supply from surrounding organs. They have a reported incidence of 0.12%–0.57% after laparoscopic surgery with power morcellation [2, 3]. Sarcomas are commonly diagnosed postoperatively as it is difficult to distinguish a uterine sarcoma from a uterine fibroid preoperatively. Neither preoperative imaging nor clinical history is reliable in the diagnosis of a uterine sarcoma. Patients who undergo hysterectomy or myomectomy for fibroids have a 0.05%–0.35% risk of an unsuspected uterine sarcoma [4]. Occult malignancy is more common than previously thought, and power morcellation poses a risk of spreading occult malignant tissue, which worsens patients' long-term survival [5].

## 2. Case Report

A 52-year-old Japanese woman complained of lower abdominal pain, constipation, and perception of an abdominal tumor for three months. She was gravida 0 and para 0. Her medical history included hypertension and schizophrenia. She had undergone an appendectomy in her childhood and total laparoscopic hysterectomy (TLH) for a leiomyoma four years previously. The uterus had weighed 398 g and two submucosal fibroids had been detected (the larger with 3 cm diameter). She had not undergone computerized tomography (CT) or magnetic resonance (MR) imaging before the initial surgery because the tumors had been small, ultrasonography had not revealed any sign of necrotizing or hemorrhagic tumor, and she had not wanted these investigations. LDH was found to be 196 U/mL at the initial visit and tumor markers were not assessed. The TLH was performed with an uncovered power morcellator to remove the tissue from the abdominal cavity. There were no immediate postoperative complications and an annual follow-up was recommended, which the patient discontinued on her own. She now presented to the internal medicine clinic, and CT revealed a large tumor in her pelvis measuring 25 cm in diameter. She was referred to our hospital for a suspected ovarian tumor.

**Figure 1:**
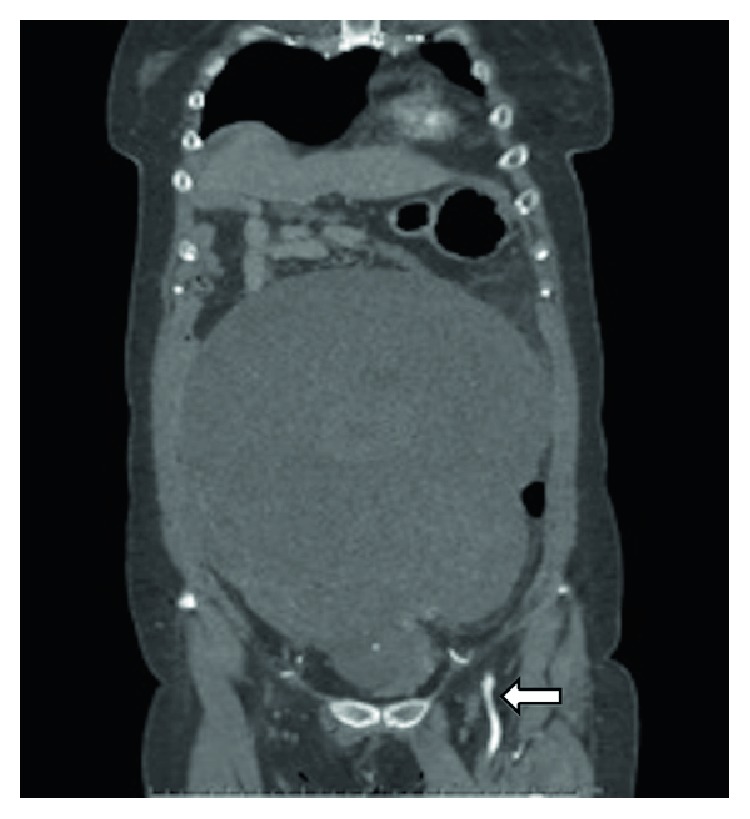
Contrast-enhanced computed tomography. The tumor received its blood supply mainly from the left internal iliac artery (arrow).

The tumor extended till the inferior border of the ensiform cartilage, and ultrasonography showed a homogeneous solid tumor like a leiomyoma. The tumor received its blood supply mainly from the left internal iliac artery, but it did not show enhancement following contrast-enhanced CT (Figure 1).

**Figure 2:**
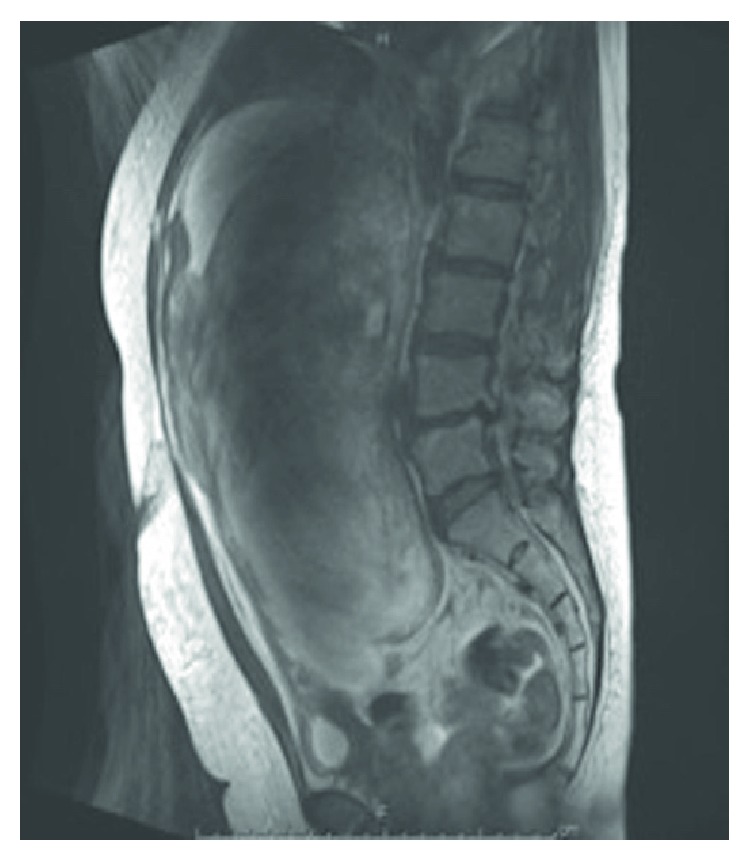
T2-weighted magnetic resonance imaging.

No tumor metastasis was detected. Contrast-enhanced MR imaging showed high-intensity signals on T2-weighted MR images, which indicated a degenerating myoma (Figure 2).

Laboratory evaluation revealed that CA125 was elevated to 361 U/mL, and LDH was within the normal range at 225 U/L. The vaginal stump cytology was negative. Edoxaban was administered for deep vein thrombosis of the left soleal vein caused by the pressure of the large tumor.

**Figure 3:**
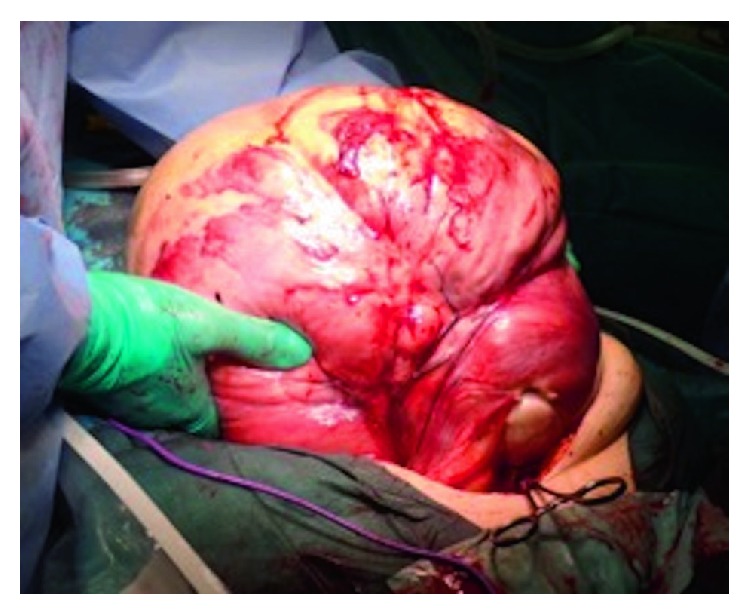
The large tumor was easily removed from the abdominal cavity.

Considering her past medical history of TLH for fibroids, a parasitic leiomyoma was diagnosed, and tumor excision and bilateral salpingo-oophorectomy were performed. There were no adhesions in the abdominal cavity except on the vaginal stump and on part of the left side of the retroperitoneum (Figure 3).

**Figure 4:**
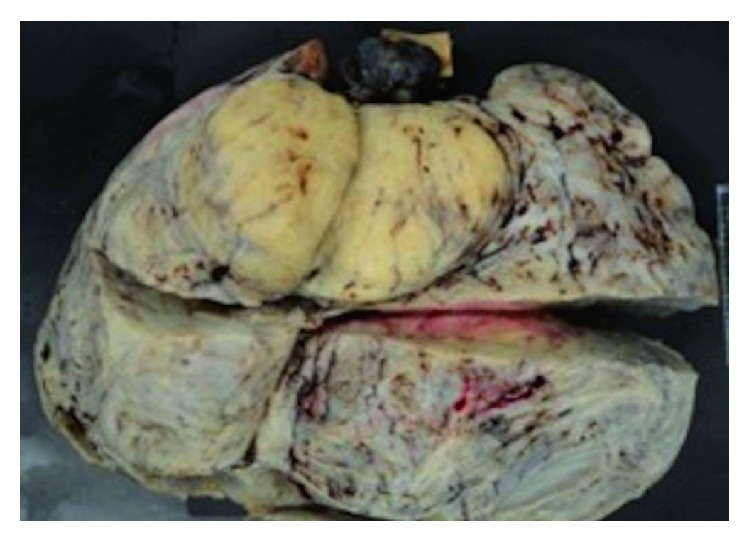
Specimen fixed with formalin.

Bilateral uterine adnexa were normal, and the tumor weight including the bilateral uterine adnexa was 7286 g. The specimen was a solid tumor that contained hemorrhagic necrosis (Figure 4).

**Figure 5:**
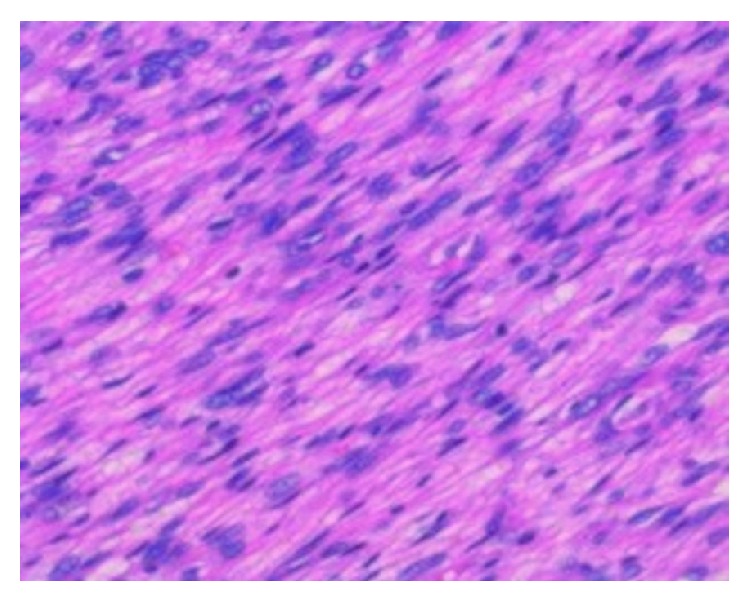
High-powered field view of hematoxylin eosin staining

Pathological evaluation revealed that the tumor comprised fascicles of spindle-shaped cells that possessed hyperchromatic fusiform nuclei. The mitotic index exceeded 10 figures per 10 high-powered fields (Figure 5).

Coagulative tumor cell necrosis and hyaline necrosis were seen. In addition, immunostaining showed diffuse and focal *α*-smooth muscle and desmin, respectively. The positive rate of Ki-67 was 33%. However, immunostaining was negative for CD34, S-100, c-kit, and DOG-1. Thus, the tumor was diagnosed as a leiomyosarcoma and not a leiomyoma. Both uterine adnexa were benign, and the ascites cytology was negative. The patient developed ileus after the surgery, but was discharged on the 15^th^ postoperative day. CA125 testing was subsequently negative. Although it was a malignant tumor, lymph node dissection was not performed as per the patient's wish. Moreover, as a benign tumor had been suspected prior to surgery, the tumor had been removed completely. She has been followed-up carefully now without any additional treatment and has been recurrence-free for 20 months postsurgery so far. Although the initial pathological evaluation was reviewed at the time of the second evaluation, malignancy was not detected.

## 3. Discussion

In this patient, an uncontained power morcellator was used at the initial surgery, which was before the U.S. Food and Drug Administration (FDA) issued the critical advisory for power morcellation [5]. FDA has warned that “women who have had fibroid surgery with a laparoscopic power morcellator were later found to have a hidden uterine sarcoma, have lower disease-free survival…, when compared to women who were treated with manual morcellation or without morcellation” [5]. In the retrospective Multicentre Italian Trialists in Ovarian Cancer and Gynecologic Malignancies group study, patients who underwent morcellation of undiagnosed leiomyosarcomas experienced a 3 times higher risk of death compared to those who had no morcellation [6]. Although in our patient, there was no suspicion of malignancy before initial surgery in terms of tumor size and character by ultrasonography and pathological evaluation revealed a leiomyoma at the initial surgery, a diagnosis of leiomyosarcoma was now made. Review of the initial specimen at the present time did not detect any malignant tissue. Tan-Kim et al. reported on the incidence of uterine sarcomas after laparoscopic hysterectomy with power morcellation [7]. The overall incidence of an occult uterine sarcoma was 6 of 941 (0.61%) in women who underwent laparoscopic hysterectomy using power morcellation. Three of the six patients were diagnosed with sarcoma on initial pathological evaluation of the morcellated specimen. However, the other three patients had delayed diagnosis of sarcoma, as the initial evaluation was benign. They had recurrence of abdominal or pelvic masses that were subsequently diagnosed as uterine sarcomas. The median amount of time between initial and second evaluation was 6 years. Two of the three patients had their initial evaluations reviewed at the time of the second evaluation. However, only one was diagnosed with sarcoma from the initial specimen and no malignancy was detected in the available specimens of the initial operation for the other patient. Our case is similar to the latter case. Power morcellation has the disadvantage of difficulty in orienting the small morcellated fragments of the specimen. [7, 8]. The morcellated specimens lack anatomic features, which increase the risk of missing the most suspicious areas during microscopic examination. Moreover, power morcellators distort normal tissues, making the diagnosis more difficult. It is also not known whether the initial disease, diagnosed as uterine fibroids, had sarcoma that was missed or whether the disseminated fibroids after the initial MIS were transformed into malignant tissue.

To prevent dissemination of specimens into the abdominal cavity following power morcellation, a novel in-bag morcellation technique for contained power morcellation has been proposed. Cohen et al. evaluated the safety of contained power morcellation [9]. Although there was dye leakage from the bag in a few cases (9.2%), the bags were intact. The leakage might have occurred at the time of bag removal or via a microperforation. The clinical significance of this leakage is unclear because open surgery was performed, which also may involve uterine disruption and fluid spread, particularly in myomectomy. The results supported the feasibility of contained power morcellation. In 2016, the FDA allowed the marketing of certain contained power morcellation systems, namely the PneumoLiner (OLYMPUS, USA) [10]. However, they warned that the containment system had not been proven to reduce the risk of dissemination of hidden malignant tissue during surgery, and, therefore it was not to be used for suspected malignant tissues. Healthcare providers should choose the best treatment approach and inform their patients of the risks of occult uterine sarcoma. When there is an alternative approach to remove even benign tumors with MIS, power morcellation should not be used, if possible. If power morcellation is necessary, the contained morcellation system should be used to minimize dissemination.

## 4. Conclusion

We encountered a case of leiomyosarcoma that occurred following laparoscopic hysterectomy with a power morcellator. Occult malignancy should be considered not only preoperatively in uterine fibroids but also after surgery with power morcellation for fibroids on the initial pathological evaluation, owing to the difficulty in diagnosis. Moreover, to prevent tissue dissemination, uncontained power morcellation should not be performed. Even if a benign tumor is suspected, contained power morcellation should be chosen carefully. Additionally, patients should be informed about the possible dissemination of tissues and occurrence of occult malignancy before they undergo power morcellation.
